# Social disparities in parental smoking and young children’s exposure to secondhand smoke at home: a time-trend analysis of repeated cross-sectional data from the German KiGGS study between 2003-2006 and 2009-2012

**DOI:** 10.1186/s12889-016-3175-x

**Published:** 2016-06-08

**Authors:** Benjamin Kuntz, Thomas Lampert

**Affiliations:** Department of Epidemiology and Health Monitoring, Robert Koch Institute, General-Pape-Straße 62-64, Berlin, 12101 Germany

**Keywords:** Secondhand smoke (SHS) exposure, Environmental tobacco smoke (ETS), Children, Parental smoking, Socioeconomic position, Health inequalities, KiGGS, Germany, Smoke-free legislation, Tobacco control

## Abstract

**Background:**

Children who are exposed to secondhand smoke (SHS) have an increased risk of a wide range of health problems and illnesses. Smoke-free legislation aims to improve indoor air quality and in this way protect the health of people who do not smoke. This paper examines trends in SHS exposure at home among children in Germany since the introduction of smoking bans in public places. Special focus is placed on the importance of the family of origin’s socioeconomic status (SES) and on parental smoking behaviour.

**Methods:**

The analyses are based on two waves of the “German Health Interview and Examination Survey for Children and Adolescents” (KiGGS)—one of which was conducted immediately before the introduction of central smoke-free legislation in the 2003-2006 period, the other approximately 6 years later from 2009 to 2012. A comparison is made between the answers given by the parents of children aged between 0 to 6 (KiGGS baseline study, *n* = 6680; KiGGS Wave 1, *n* = 4455). Domestic SHS exposure is covered in the parent interviews by asking whether anyone is allowed to smoke at home in the presence of their child. Parental smoking behaviour is determined separately for mothers and fathers. SES is determined on the basis of the parents’ education, occupational status and income.

**Results:**

The percentage of 0- to 6-year-old children exposed to SHS in the parental home fell from 23.9 to 6.6 % in the period from 2003-2006 to 2009-2012. At the same time, the percentage of children with at least one parent who smokes decreased from 49.8 to 41.8 %. While relative social inequalities in parental smoking behaviour have tended to increase over time, inequalities in domestic SHS exposure have persisted. Children whose parents smoke and children from low-SES families are still most likely to be exposed to tobacco smoke. In both study periods and after statistical adjustment for parental smoking behaviour, children with a low SES had a 6.6-fold higher risk for SHS exposure in the parental home than children from high-SES households.

**Conclusions:**

The results of the KiGGS study show that the proportion of children in Germany who are exposed to SHS at home has declined significantly over the last few years. There is much to suggest that the smoke-free legislation that has been introduced in Germany has led to a heightened awareness of the health risks of SHS both in public and in the private sphere, as well as to a denormalization of smoking. Children whose parents smoke, and among them particularly children from socially disadvantaged families, should be recognised as key target groups when implementing future tobacco-control measures.

## Background

Tobacco smoke is the most important avoidable indoor air pollutant [1, 2]. In 2004 an estimated 600,000 people worldwide died of diseases caused by prolonged exposure to secondhand smoke (SHS) [3]. About 28 % of these deaths occurred in children under the age of 15 [3]. Cases of Sudden Infant Death Syndrome (SIDS), of which SHS exposure is a key risk factor [4, 5], were even not included in this study [3]. Children who are regularly exposed to SHS are more likely to suffer from respiratory complaints, asthma and infections of the lower respiratory tract [4, 6–9]. Middle-ear infections are also more prevalent among SHS-exposed children [10]. Furthermore, children whose parents or siblings smoke are more likely to start smoking as adolescents [11]. The diseases and deaths caused by SHS generate high economic costs and health-sector expenditure [12–14].

In the case of children, it must also be taken into account that they have a higher respiratory rate than adults, that their organs react particularly sensitively to environmental pollutants such as tobacco smoke during growth and maturation processes, and that their body’s detoxification system is not yet fully developed [4, 7]. Whereas adult non-smokers can at least to some extent ensure a smoke-free environment for themselves, children are frequently exposed to SHS with no protection [15]. In addition, compared to adolescents and adults, children have limited control over their indoor environment and spend much of their time in closed rooms [16]. Private homes, and above all the parental home, are therefore the main places where children are exposed to SHS [3, 15, 17, 18].

In order to offer both children and non-smoking adolescents and adults better protection from the health risks of SHS, far-reaching laws to protect non-smokers have been introduced in recent years in Germany and in most other European countries [19–21]. An essential point of reference in this context is Article 8 of the World Health Organization’s Framework Convention on Tobacco Control (FCTC), which calls for the comprehensive protection of the population against SHS [22, 23]. After changes to Germany’s Workplace Ordinance led to extensive bans on smoking at the workplace in 2004, nationwide smoking bans in public buildings, railway stations and on public transport came into force in 2007. Laws to protect non-smokers were enacted in Germany’s 16 federal states in 2007 and 2008, prohibiting smoking in public amenities, in healthcare, cultural, sporting and educational facilities, as well as in catering establishments. Exceptions for pubs, restaurants and cafés, however, still stand in the way of a comprehensive protection of non-smokers in most of Germany’s states [24].

The smoking bans implemented as part of legislation to protect non-smokers have indeed led to a marked reduction in SHS exposure in public spaces and to a fall in the prevalence of SHS-associated diseases [25–27]. The effects on the unregulated private sphere are less unequivocal. The impact of smoke-free legislation on adult smoking behaviour on private premises—and thus also on the exposure of the non-smoking population to SHS on those premises—is the subject of controversial discussion. The *displacement hypothesis* (or *last refuge model*) postulates that it leads to a kind of evasive manoeuvre, i.e. the more smoking is banned in public, the more people will smoke at home [28–30]. By contrast, the *social diffusion hypothesis* holds that smoking bans in public places also have a knock-on effect on the private sphere. If non-smoking increasingly becomes the social norm and there is a greater overall awareness of the health risks of smoking and SHS, then more smokers will be encouraged to give up smoking altogether, or at least to cut it down, in the non-regulated private sector too [28, 29]. In line with health behaviour change theories and models (e.g. Bronfenbrenner’s socio-ecological model or the Behavioral Ecological Model (BEM)), both hypotheses acknowledge that environmental factors and social norms might influence parental smoking behaviour and thus children’s SHS exposure in the home [31, 32].

According to studies, parental smoking behaviour and the family’s socioeconomic position are the two key determinants when it comes to the domestic exposure of children to SHS [33–37]. Children whose parents smoke are also disproportionately more frequently exposed to SHS at home than the children of non-smoking parents [35, 36]. When indicators of parental socioeconomic status (SES) are taken into consideration, it transpires that children from socially disadvantaged families are exposed to SHS much more frequently than children from families that are socially better off [33–35, 38, 39]. Social inequalities in parental tobacco use could partly explain SES differences in children’s SHS exposure at home. In Germany, as in many other high income countries, smoking is more prevalent among lower SES groups [40–42].

This paper examines the development of levels of domestic SHS exposure among children in Germany after the introduction of smoke-free legislation in public places. It analyses both the importance of SES and the influence of parental smoking behaviour—as well as their mutual interactions—on SHS exposure over time. The aim is to find answers to the following four research questions:

1. How did the domestic SHS exposure of 0- to 6-year-old children develop in the period from 2003-2006 to 2009-2012?
2. How great is the influence of parental smoking behaviour on the SHS exposure of children?
3. To what extent are there social disparities in parental smoking behaviour and in SHS exposure among children, and what changes have taken place in these areas over time?
4. To what extent can parental smoking behaviour help explain social disparities in SHS exposure among children?

## Methods

### Study population

The “German Health Interview and Examination Survey for Children and Adolescents” (KiGGS) is part of the health monitoring system run on behalf of the German Federal Ministry of Health by the Robert Koch Institute (RKI), the national public health institute. The KiGGS study is a combined cross-sectional and cohort study; its objectives, concept and design are described in detail elsewhere [43–46]. KiGGS aims to regularly provide prevalence data collected nationwide on the health situation of children and adolescents living in Germany, focusing on the 0–17 age group. The KiGGS baseline study (2003-2006) comprised interviews, physical examinations and laboratory analyses; the first follow-up survey, KiGGS Wave 1 (2009-2012), was carried out as a telephone-based survey. The KiGGS baseline study was a pure cross-sectional study with a total of 17,641 subjects aged between 0 and 17; the response rate was 66.6 %. Those invited to participate were randomly drawn from the population registers in a stratified random sample of 167 locations in Germany [44]. The sample of KiGGS Wave 1 consisted firstly of a new cross-section sample of 0- to 6-year-olds who were again drawn at random from the population registers of the original study locations. Secondly, the former participants in the KiGGS baseline study were invited to take part in the new survey (KiGGS cohort). A total of 12,368 children and adolescents in the 0–17 age range relevant for the cross-section took part; 4455 of these were invited for the first time (response 38.8 %), and 7913 were invited again (response 72.9 %) [46]. In this paper, time-trend analysis is solely based on repeated cross-sectional data.

### Ethical considerations

Before the study began, votes of approval had been obtained from the Ethics Commission of Charité University Hospital Berlin, and Germany’s Federal Commissioner for Data Protection; an interview was only carried out after either the subjects themselves (in the case of adults) or the persons having care and custody (in the case of minors) had been informed and had given their consent in writing.

### Domestic exposure to secondhand smoke

The analyses on domestic SHS exposure are limited to the survey replies of the parents of children aged 0 to 6 years. The information on 6680 children from the KiGGS baseline study and the data of 4455 boys and girls from KiGGS Wave 1 are analysed (Table 1). The question asked was: “Is anybody allowed to smoke at home in the presence of your child and, if so, how often?” (answer categories: “Daily”, “Several times a week”, “Once a week”, “Less often”, “Never”). For this paper, the first four answer categories indicating regular or at least occasional exposure to SHS will be treated as one [47]. No modifications were made in either the question or the default answer categories between the KiGGS baseline study and KiGGS Wave 1. This means that, allowing for the changed survey mode (written vs. telephone survey), statements can be made on the development of domestic SHS exposure over time.

**Table 1:** Characteristics of the KiGGS study population with regard to children aged 0 to 6 years

		KiGGS baseline study (2003-06)		KiGGS Wave 1 (2009-12)	
		(*n* = 6680)		(*n* = 4455)	
		n^a^	%^b^	n^a^	%^b^
Age (years)	0	935	11.6	634	11.6
	1	925	14.4	641	14.3
	2	945	14.8	667	14.8
	3	934	14.8	601	14.8
	4	982	14.5	663	14.5
	5	953	14.8	633	14.8
	6	1006	15.1	616	15.2
Sex	Boys	3367	51.3	2290	51.3
	Girls	3313	48.7	2165	48.7
Socioeconomic status (SES)	Low	1023	19.6	359	17.5
	Medium	3926	58.4	2685	59.2
	High	1654	22.0	1409	23.3
	Missings	77	-	2	-
Domestic secondhand smoke exposure	Daily	465	8.9	52	2.4
	Several times a week	155	2.8	10	0.2
	Once a week	46	0.7	16	0.3
	Less often	686	11.4	109	3.6
	Never	5210	76.1	4266	93.4
	Missings	118	-	2	-
Paternal smoking behaviour	Yes	2593	42.0	1236	34.7
	No	3778	58.0	2914	65.3
	Missings	309	-	305	-
Maternal smoking behaviour	Yes	1932	31.0	890	25.1
	No	4661	69.0	3555	74.9
	Missings	87	-	10	-
Parental smoking behaviour	Both parents smoke	1256	21.0	484	15.4
	One parent smokes	1809	28.7	1019	26.4
	Neither parent smokes	3273	50.2	2638	58.2
	Missings	342	-	314	-

^a^Unweighted

^b^Percentages were calculated without missing values and weighted with regard to age, gender, region, nationality, type of municipality, and the education status of the head of the household (population structure in Germany 2009/2010)

### Parental smoking behaviour

The interviews of the parents also collected information on the current smoking behaviour of both parents. The question asked was whether the father or mother currently smokes (answer categories: “Yes, daily”, “Yes, sometimes”, “No”), although no distinction is made between daily and occasional tobacco use in the following (Table 1). In order to investigate any cumulative effect of parental smoking behaviour on domestic SHS exposure, a new three-stage variable was formed from the information on the smoking behaviour of the mother and the father (categories: “Neither parent smokes”, “One of the parents smokes”, “Both parents smoke”). Children for whom valid data were not available on both parents’ current smoking behaviour were excluded from the analyses on parental smoking behaviour (KiGGS baseline study: *n* = 342, KiGGS Wave 1: *n* = 314).

### Socioeconomic status (SES)

SES is determined on the basis of an index developed by the RKI and frequently used in population-based studies in Germany [48–50]. This index contains information provided by the parents on their school education and vocational training, their occupational status and their income, making it possible to classify them in a low-, middle- or high-status group (Table 1). The individual dimensions of education and occupation were collected separately for each parent and the higher value used respectively for the overall index. Income was recorded as a characteristic of the household; the net equivalent income was calculated based on the number and age of the household members. Missing data on income were imputed using a regression model. In order to create the index, the status characteristics were first transformed into three metric subscales with a value range from 1.0 to 7.0. Then the point scores of the subscales were added to make a total score with a value range from 3.0 to 21.0. The classification into a low-, middle- or high-status group is based on a distribution-based definition of five groups with equal numbers of members (quintiles); the middle three groups (from 2nd to 4th quintile) are combined. Detailed information on the measurement of SES in the KiGGS study has been published elsewhere [48].

### Statistical analyses

All analyses were conducted with a weighting factor that corrects the sample’s deviations from the population structure (figures for 31 December 2010) with regard to age, gender, region, nationality, type of municipality, and the education status of the head of the household [46]. Reports are made on prevalences with 95 % confidence intervals, taking differences in SES and parental smoking behaviour into account. With a view to possible differences in the distribution of domestic SHS exposure, odds ratios (OR) are also reported; these were calculated using binary logistic regressions. Odds ratios indicate the factor by which the statistical chance of domestic SHS exposure is increased in children with a low SES or with parents who smoke, compared to children with a high SES or with non-smoking parents respectively.

In order to take into account both the weighting and the correlation of the participants within a municipality, the confidence intervals and p-values were calculated using procedures for complex samples. Group differences were checked for significance according to Rao-Scott using the chi-square test for complex samples corrected via the F distribution [51]. Differences are regarded as statistically significant if the confidence intervals do not overlap or if the probability of error (p) takes on a value smaller than 0.05. All the analyses were performed using the IBM SPSS Statistics Version 20 software product.

## Results

**Fig. 1:**
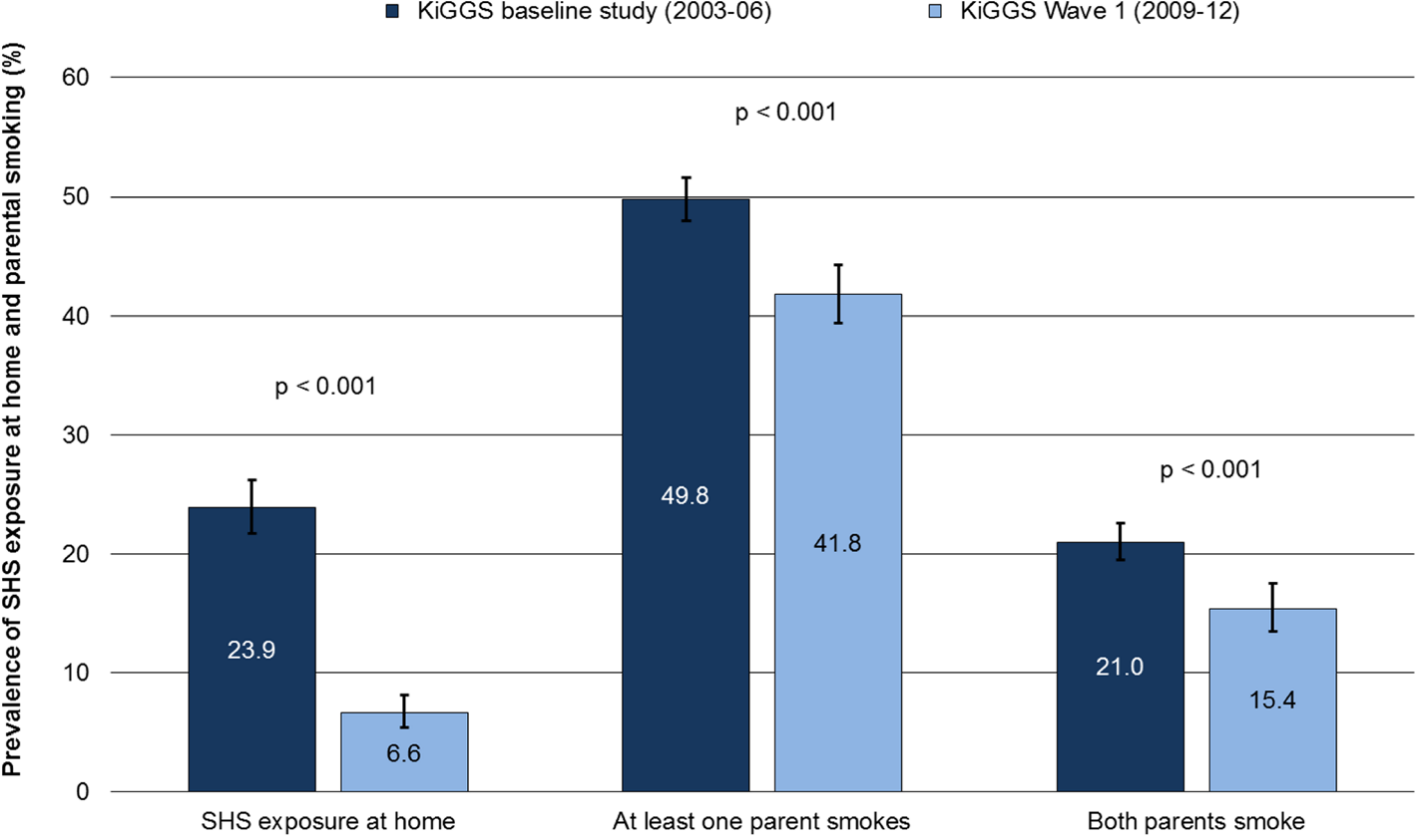
Secondhand smoke (SHS) exposure at home and parental smoking behaviour among 0- to 6-year-old children in Germany

The percentage of 0- to 6-year-old children who are exposed to SHS in the parental home fell from 23.9 to 6.6 % in the period from 2003-2006 to 2009-2012. The proportion of children with parents who smoke also decreased — although not by quite as much. According to the data from KiGGS Wave 1, 41.8 % of children in 2009-2012 had at least one parent who smoked (down from 49.8 % in 2003-2006), and both parents smoked in the case of 15.4 % (2003-2006: 21.0 %) of children (Fig. 1). As Table 1 shows, the percentage of both fathers and mothers who smoke has declined over time.

Children whose parents smoke are significantly more affected by SHS exposure than children whose parents do not smoke. In the 2003-2006 period, 52.3 % of children with two smoking parents, 30.2 % of children with one smoking parent, and 5.6 % of children with no smoking parent were exposed to tobacco smoke at home. In the 2009-2012 period, the corresponding percentage in each of the three groups was considerably lower at 14.9, 10.2 and 1.6 % respectively. The relative decline in SHS exposure was thus about the same for children of smoking parents as for children whose parents do not smoke.

**Fig. 2:**
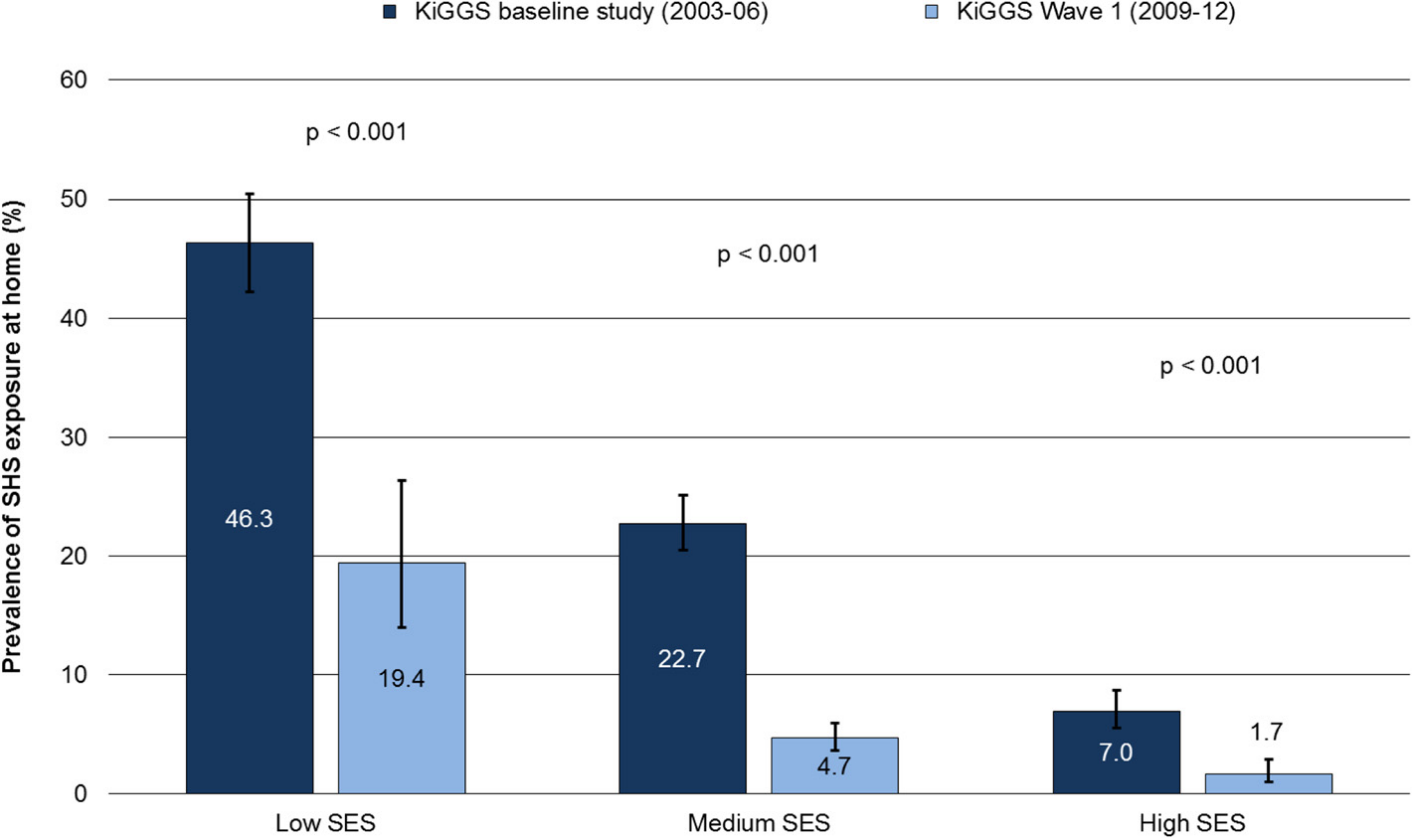
Secondhand smoke (SHS) exposure at home by socioeconomic status (SES) among 0- to 6-year-old children in Germany

**Fig. 3:**
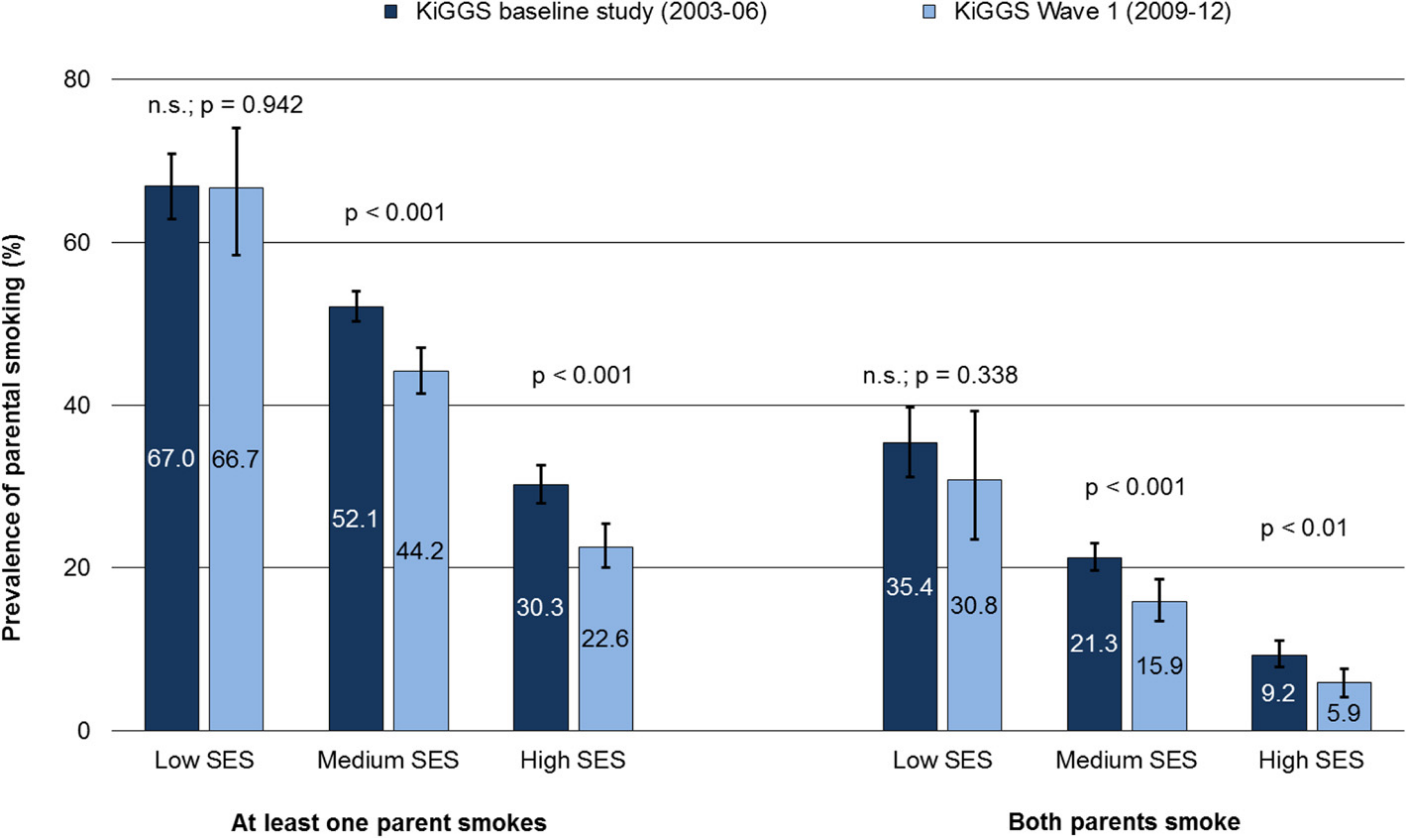
Parental smoking behaviour by socioeconomic status (SES) among 0- to 6-year-old children in Germany

Children with a low SES are more frequently exposed to SHS in the parental home and more likely to have parents who smoke than children with a high SES (Figs. 2 and 3). However, declining prevalences in domestic SHS exposure can be observed in children of all status groups (Fig. 2). On the other hand, the proportion of children whose parents smoke has only decreased to a statistically significant extent among those from the middle- and high-status groups (Fig. 3). As a result, at least the relative social inequalities have increased over time.

**Fig. 4:**
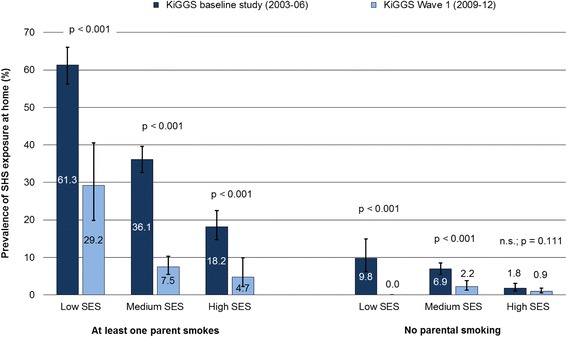
Secondhand smoke (SHS) exposure at home by socioeconomic status (SES) among 0- to 6-year-old children in Germany, stratified by parental smoking behaviour

When stratified according to parental smoking behaviour, the analyses indicate that, according to the data from KiGGS Wave 1, social disparities in the levels of SHS exposure only still apply to children whose parents smoke (Fig. 4). In the KiGGS baseline study there had also been a marked social gradient in domestic SHS exposure among children whose parents do not smoke, meaning that household and family members or visitors were much more likely to be allowed to smoke — even in the presence of the children — in the homes of socially disadvantaged parents than in the homes of parents who were socially better off (9.8 vs. 1.8 %). In the KiGGS baseline study, of all children with smoking parents those with a low SES were exposed much more often to SHS in the parental home than children with a middle SES, and the latter, in turn, more often than children with a high SES; in KiGGS Wave 1, by contrast, significant disparities only existed between children with a low and high SES and between children with a low and middle SES. In the case of children with at least one smoking parent, although the percentage of those who are exposed to SHS at home has also more than halved in the low-status group over time, the relative decline in SHS exposure was even more pronounced in the middle- and high-status groups (Fig. 4).

As the multivariate analysis shows, both SES and parental smoking behaviour are associated with the SHS exposure of 0- to 6-year-old children — each factor independently of each other (Table 2). A comparison of the two analytical models reveals that the association between SES and the domestic exposure of children to SHS is reduced by about half when parental smoking behaviour is taken into account. Vice versa, the SES explains about a quarter of the association between parental smoking behaviour and domestic SHS exposure among children. Even taking into account the fact that children from socially disadvantaged families are more likely to have parents who smoke (Fig. 3), the data from KiGGS Wave 1 indicate that children with a low SES have a 6.6-fold higher risk for SHS exposure in the parental home than children with a high SES. Even children with a middle SES still have a 1.7-fold higher risk, although this is not statistically significant. The corresponding odds ratios were 6.6 and 2.8 respectively in the KiGGS baseline study. Vice versa, after statistical adjustment for SES, a child is about 5.6 and 7.4 times more likely to be exposed to SHS at home if one or both parents smoke than if neither of them smoke. In the KiGGS baseline study, the corresponding odds ratios were even higher at 6.5 and 15.3 respectively.

**Table 2:** Domestic secondhand smoke (SHS) exposure among 0- to 6-year-old children in Germany by socioeconomic status (SES) and parental smoking behaviour

	KiGGS baseline study (2003-2006)		KiGGS Wave 1 (2009-2012)	
	Model 1	Model 2	Model 1	Model 2
	OR (95 % CI)	OR (95 % CI)	OR (95 % CI)	OR (95 % CI)
	*p* value	*p* value	*p* value	*p* value
Socioeconomic status (SES)				
Low	**11.63 (9.11–14.85)**	**6.58 (4.97–8.71)**	**13.59 (7.03–26.28)**	**6.59 (3.17–13.68)**
	*p* < 0.001	*p* < 0.001	*p* < 0.001	*p* < 0.001
Medium	**3.93 (3.11–4.96)**	**2.76 (2.10–3.62)**	**2.77 (1.54–5.00)**	1.73 (0.94–3.20)
	*p* < 0.001	*p* < 0.001	*p* < 0.001	*p* = 0.080
High	Ref.	Ref.	Ref.	Ref.
Parental smoking behaviour				
Both parents smoke	**19.10 (15.38–23.73)**	**15.29 (12.21–19.13)**	**11.08 (5.90–20.80)**	**7.40 (3.78–14.48)**
	*p* < 0.001	*p* < 0.001	*p* < 0.001	*p* < 0.001
One parent smokes	**7.43 (5.98–9.23)**	**6.53 (5.21–8.17)**	**7.08 (4.01–12.50)**	**5.55 (3.16–9.75)**
	*p* < 0.001	*p* < 0.001	*p* < 0.001	*p* < 0.001
Neither parent smokes	Ref.	Ref.	Ref.	Ref.

Model 1: adjusted for age and sex of the child, Model 2: + mutually adjusted for SES and parental smoking behaviour; bold = significant at .05 level; Results of the KiGGS baseline study and KiGGS Wave 1 were adjusted to the population structure in Germany 2009/2010

## Discussion

The results of the KiGGS study show that the percentage of 0- to 6-year-old children in Germany who are exposed to SHS in the parental home has declined by more than half over the last few years. This is all the more remarkable in view of the fact that the proportion of children whose parents smoke has not fallen to the same extent. It seems that both non-smoking parents and parents who themselves smoke are increasingly making sure that no one smokes in the presence of their children at home.

Our findings are in line with international study results on the development of children’s domestic exposure to SHS over time. Current studies from the UK [18, 52–54], Denmark [34], the USA [55, 56], Australia [57] and Japan [38] indicate that the percentage of children who are exposed to SHS at home has decreased noticeably over the last few years, in some cases immediately after the introduction of laws to protect non-smokers. In a comparison of 3 years of school enrolment — 2004/2005, 2005/2006 and 2008/2009 — regional study results in Bavaria show that the percentage of 5- to 6-year-old children from homes where people smoke has at least not increased [17]. The available results thus indicate that the introduction of smoking bans in public places has not led to people smoking more at home, as some experts had feared [30]. Rather, the findings can be interpreted according to the *social diffusion hypothesis*, which states that smoke-free legislation can also have positive effects on hitherto unregulated areas such as private households [28, 29].

The KiGGS results confirm for both study periods the finding (which is well known from literature) that parents who smoke must be regarded as the main source of domestic SHS exposure among children [35, 36]. Whereas more than half of children whose parents both smoked were exposed to SHS at home in 2003-2006, this figure had fallen to about 15 % by 2009-2012. In the case of children whose parents are both non-smokers, the already small proportion of those who are exposed to SHS at home (e.g. by the tobacco consumption of other household members or visitors) also fell significantly from 5.6 to 1.6 %.

That children from socially disadvantaged families have a higher risk of exposure to SHS at home has been documented several times both in Germany [33, 58–61] and internationally [35, 38, 39, 52, 54, 57, 62, 63]. As our analyses show, the relative decline in SHS exposure has been equally strong in all status groups, i.e. there has been no increase in relative social inequalities in the domestic exposure of children to SHS. This result is consistent with the findings of a regional study in Bavaria [17] and several international studies [53, 54]. However, other studies document an increase in relative social inequalities in the field of SHS exposure among children [38, 57]. According to the KiGGS data, there has been an increase in the relative social inequalities relating to parental smoking behaviour, since the percentage of children whose parents smoke has only declined to a statistically significant extent in the middle- and high-status groups. Our results thus confirm study findings from Germany and other industrialized countries, most of which consistently show that socioeconomic inequalities in smoking among adults have increased over the last 20 years [41, 64–67]. Social disparities in smoking behaviour and in SHS exposure can be found in different age and population groups. Already published results of the KiGGS study suggest that significant social disparities also exist in relation to maternal smoking during pregnancy [68] as well as in daily tobacco consumption and in the SHS exposure of 11- to 17-year-old adolescents [69].

### Limitations

Our results are based on a nationwide, representative data basis. The informative value of the findings is, however, limited by the fact that the data on parental smoking behaviour and children’s SHS exposure in the parental home are based on information provided by the respondents themselves. Socially desired response behaviour — leading to lower figures for the actual percentage of parents who smoke and for the number of children who are exposed to SHS—cannot therefore be ruled out (*social desirability bias*). In KiGGS Wave 1 it was not possible to carry out measurements of air pollutant levels in rooms or to take samples to determine levels of cotinine (a metabolite of nicotine) in children’s saliva, urine or blood—as have been carried out in other studies [39, 54–56, 61, 62] to quantify the true levels of SHS exposure.

As described in the methods section, children for whom valid data were not available on both parents’ current smoking behaviour were excluded from all the analyses in which parental smoking behaviour was considered—as in a similar study [38]. However, these are often children of single parents (usually mothers). Since it is known from literature that an above-average number of single parents are smokers [70–72], we conducted additional analyses in which the family type was taken into account (results not shown). In line with current research, these revealed that the 0- to 6-year-old children of single parents are more frequently exposed to SHS at home than peers who live in two-parent families (KiGGS baseline study: 44.5 vs. 22.0 %; KiGGS Wave 1: 13.4 vs. 5.9 %). This is most likely due to the fact that it is rarer for children who grow up in two-parent families to have smoking mothers than children of single parents (KiGGS baseline study: 28.3 vs. 57.8 %; KiGGS Wave 1: 22.7 vs. 46.9 %). Yet here, too, there are declining prevalences in both groups, despite the differences between the groups.

Finally, the change in the survey mode that took place between the KiGGS baseline study and KiGGS Wave 1 must also be taken into account [46]. In the baseline study, the data on parental smoking behaviour and SHS exposure in the parental home were collected using self-completed questionnaires; by contrast, computer-aided telephone interviews were used in the follow-up survey. Since the tendency towards social desirability has occasionally been seen to be greater in interviews than in written surveys [73, 74], the possibility cannot be excluded that the downward trend in parental smoking behaviour and domestic SHS exposure among children might be based at least partially on a “mode effect”. Whether such an effect does indeed exist, and how parental smoking behaviour and domestic SHS exposure among children will develop in the future, cannot be estimated on the basis of the KiGGS data until 2017 at the earliest. Written questionnaires will again be used in KiGGS Wave 2, whose field phase will last about 3 years (2014-2017) [75].

## Conclusions

In Germany, the prevalence of SHS exposure at home among children has declined markedly over the last few years. The percentage of children whose parents smoke has also fallen. There is much to suggest that the smoke-free legislation that has been introduced in Germany has led to a heightened awareness of the health risks of SHS both in public and in the private sphere, as well as to a denormalization of smoking. While relative social inequalities in parental smoking behaviour have tended to increase over time, inequalities in domestic SHS exposure have persisted. Children whose parents smoke — and among them in particular children from socially disadvantaged families — should be recognised as key target groups when implementing future tobacco-control measures.

## Abbreviations

FCTC, World Health Organization’s Framework Convention on Tobacco Control; KiGGS, German Health Interview and Examination Survey for Children and Adolescents; RKI, Robert Koch Institute; SES, socioeconomic status; SHS, secondhand smoke; SIDS, Sudden Infant Death Syndrome
